# Case Finding of Mild Cognitive Impairment and Dementia and Subsequent Care; Results of a Cluster RCT in Primary Care

**DOI:** 10.1371/journal.pone.0156958

**Published:** 2016-06-16

**Authors:** Pim van den Dungen, Eric P. Moll van Charante, Peter M. van de Ven, Harm W. J. van Marwijk, Henriëtte E. van der Horst, Hein P. J. van Hout

**Affiliations:** 1 Department of General Practice and Elderly Care Medicine, EMGO Institute for Health and Care Research, VU University Medical Center Amsterdam, Amsterdam, The Netherlands; 2 Department of General Practice, Academic Medical Center, University of Amsterdam, Amsterdam, The Netherlands; 3 Department of Epidemiology and Biostatistics, VU University Medical Center Amsterdam, Amsterdam, The Netherlands; 4 Primary Care Research Centre, Institute of Population Health, University of Manchester, Manchester, United Kingdom; Cardiff University, UNITED KINGDOM

## Abstract

**Purpose:**

Despite a call for earlier diagnosis of dementia, the diagnostic yield of case finding and its impact on the mental health of patients and relatives are unclear. This study assessed the effect of a two-component intervention of case finding and subsequent care on these outcomes.

**Methods:**

In a cluster RCT we assessed whether education of family physicians (FPs; trial stage 1) resulted in more mild cognitive impairment (MCI) and dementia diagnoses among older persons in whom FPs suspected cognitive decline and whether case finding by a practice nurse and the FP (trial stage 2) added to this number of diagnoses. In addition, we assessed mental health effects of case finding and subsequent care (trial stage 2). FPs of 15 primary care practices (PCPs = clusters) judged the cognitive status of all persons ≥ 65 years. The primary outcome, new MCI and dementia diagnoses by FPs after 12 months as indicated on a list, was assessed among all persons in whom FPs suspected cognitive impairment but without a formal diagnosis of dementia. The secondary outcome, mental health of patients and their relatives, was assessed among persons consenting to participate in trial stage 2. Trial stage 1 consisted of either intervention component 1: training FPs to diagnose MCI and dementia, or control: no training. Trial stage 2 consisted of either intervention component 2: case finding of MCI and dementia and care by a trained nurse and the FP, or control: care as usual.

**Results:**

Seven PCPs were randomized to the intervention; eight to the control condition. MCI or dementia was diagnosed in 42.3% (138/326) of persons in the intervention, and in 30.5% (98/321) in the control group (estimated difference GEE: 10.8%, OR: 1.51, 95%-CI 0.60–3.76). Among patients and relatives who consented to stage 2 of the trial (n = 145; 25%), there were no differences in mental health between the intervention and control group.

**Conclusions:**

We found a non-significant increase in the number of new MCI diagnoses. As we cannot exclude a clinically relevant effect, a larger study is warranted to replicate ours.

**Trial Registration:**

Nederlands Trial Register NTR3389

## Introduction

The diagnosis of dementia in primary care is often rather late and patients and relatives may experience obtaining a diagnosis as a struggle [1,2]. It was shown that both clinicians and patients experience avoidance in relation to the diagnosis of dementia [3]. International dementia guidelines are inconsistent regarding how physicians should proceed when they suspect mild cognitive impairment (MCI) [4–6]. However, physicians may be missing opportunities to provide psychoeducation and support for people with MCI and their carers [7–13]. In this study we assess whether case finding of MCI and dementia and subsequent care are beneficial. Case finding was defined as screening of a selected high-risk group instead of the entire group, implying an increased prior probability of disease, with the aim to bring them to ‘treatment’ [14].

There are several arguments against case finding: an increased number of diagnoses of MCI with prognostic uncertainty [15]; an increased risk of false positive diagnoses [16]; the risk of stigmatization, social isolation and losing perspective and not wanting to live anymore when diagnosed with dementia [17–21]; negative effects on the patient-physician relationship [22], and finally; the lack of a cure for dementia.

However, eight out of ten dementia patients say that they want to be informed about their diagnosis. Although often stressful, disclosure does usually not induce depression, may reduce anxiety and may even improve quality of life [23–26]. In addition, in contrast with some clinicians’ presumptions, it can end an uncertain period and mark the beginning of acceptance and adaptation for patients and caregivers [27,28].

Moreover, despite the lack of a cure for dementia, persons with dementia and their caregivers may benefit from several interventions: psychoeducation has positive short- and long-term effects on the wellbeing of both [29]. Other psychosocial interventions (such as psychoeducation, cognitive stimulation and case management) can improve patient cognitive function, behavioral problems and caregivers’ sense of competence to provide care, depressive symptoms and quality of life [29–33]. Multi-component psychosocial interventions can delay admission to a care home [29,30,34,35]. Finally, anti-Alzheimer drugs may temporarily and modestly delay cognitive and functional decline although side effects are frequent [36].

The etiology and consequences of MCI are often unclear to people, and they may interpret their symptoms as age related [37]. Still, the loss of cognitive functions may induce stress and negative emotions [38–40]. Reactions to the diagnosis range from fear of advancement to dementia to relief because dementia is not present [38]. Relatives of persons with MCI may experience a burden and are at higher risk of depressive symptoms. Roles may change and the relation with the patient may worsen. Relatives report a need for information about medical aspects of MCI and an increased need for support and services and a recent study suggests that, similar to dementia, diagnosing MCI can mark the beginning of a period of adaptation for caregivers [12,13].

A previous case finding experiment, using risk factors derived from electronic medical records combined with a subsequent cognitive assessment by telephone helped to identify a group with a higher risk of dementia and a higher use of care [41]. Subsequent diagnostic evaluation resulted in an increased number of dementia diagnoses [41]. However, the number needed to screen was high. Other studies suggested that physician education and collaboration with primary care nurses and specialized geriatric nurses may increase the rate of (suspected) dementia diagnoses, and possibly reduce the referral rate [42–44]. In one study, it also improved dementia guideline adherence [44].

So far, no studies assessed how case finding of MCI and dementia and subsequent diagnostic evaluation impact on the mental health of patients and informal caregivers. The U.S. Preventive Services Task Force concludes that the current evidence is insufficient to assess the balance of benefits and harms of screening for cognitive impairment [45]. In a cluster RCT we assessed whether education of FPs (trial stage 1) resulted in more MCI and dementia diagnoses among all older persons in whom FPs suspected cognitive decline and whether case finding and care by a practice nurse and the FP (trial stage 2) added to this number of diagnoses. In addition, we assessed mental health effects of case finding and care (trial stage 2). We included MCI as a target of case finding since we hypothesized that objectifying the impairment might have positive psychological effects caused by reassurance that it is not dementia and the provision of information and support.

## Methods

Ethical approval for the study was obtained from the medical ethics committee of the VU University Medical Center Amsterdam, The Netherlands (reference number 2010/297). The study protocol is in accordance with the principles of the current version of the declaration of Helsinki. Written informed consent was obtained from all stage 2 study participants.

### Design

**Fig 1:**
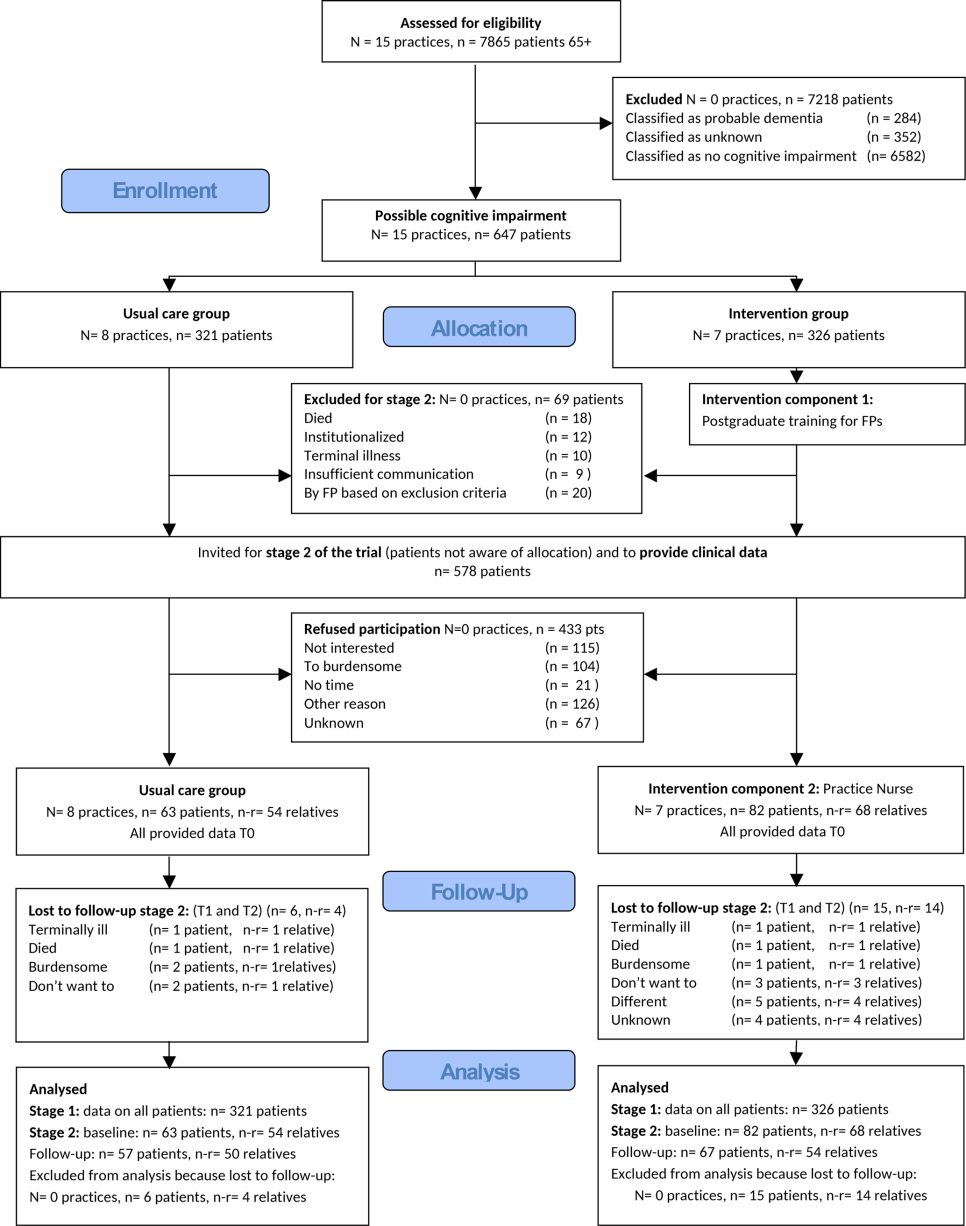
CONSORT Flowdiagram.

We performed a cluster RCT with randomization at practice level (follow-up at 6 and 12 months). All FPs in intervention practices were trained to diagnose dementia; FPs in the control practices offered usual care (stage 1 trial). All older persons with possible cognitive decline but without a formal diagnosis of dementia were included in stage 1 of the trial. Persons who had consented to participate in stage 2 of the trial received an assessment by a practice nurse (PN) and if necessary, further evaluation by the FP and subsequent care if they were registered in an intervention practice; they received usual care if they were registered in a control practice. Fig 1 provides an overview of the trial.

### Randomization

We applied a stratified randomization of the practices based on random numbers supplied by a statistician (PV), securing equal distribution of: 1) practices already working with a practice nurse (PN) providing care for frail older persons (present in 9/15 practices; on average 3.6 hrs/week); 2) percentages of patients aged 65 years and older within practices.

### Participants (stage 1 and stage 2) and setting

The primary outcome, new MCI and dementia diagnoses after 12 months, was assessed (anonymous data) among all persons (≥ 65 years) in whom FPs suspected cognitive impairment but who had no formal diagnosis of dementia. FPs performed this classification of cognition based on their recollection of clinical patient contacts and on information in the electronic medical records. As described in a previous paper, this yielded a selection of persons with a prior chance of cognitive impairment of 47.1% (according to the CAMCOG), which is substantially higher than in the general population of this age [46].

All patients were invited by letter to participate in stage 2 of the trial. The secondary outcome, mental health, was assessed among patients and relatives consenting to further assessment and care (stage 2 of the trial).

Exclusion criteria for stage 2 of the trial were:

∎ Terminal illness of the patient or informal caregiver;
∎ Permanent admission to a nursing home expected within 6 months;
∎ Insufficient understanding of spoken Dutch or incapable to express oneself.

Twenty-nine FPs working in 15 PCPs participated. The PCPs were based in two towns (27,000 and 30,000 inhabitants) near Amsterdam, The Netherlands.

### Intervention

The first component of the intervention consisted of a two-evening accredited postgraduate training of FPs. The first evening, FPs were made more aware of their own barriers (e.g. limited confidence in diagnostic skills, presumptions of harming the patient) to diagnose dementia and learned how to recognise and how to diagnose dementia according to the dementia guideline of the Dutch College of General Practitioners [4]. In addition, they were taught to diagnose MCI. The differential diagnosis, initial management, and pharmacological treatment of behavioural problems were addressed. This two and a half hour training was led by a FP who performed her PhD research on this topic and was involved in the development of the dementia guideline of the Dutch College of General Practitioners [4,44]. The second two and a half hour evening focused on performing a geriatric needs assessment, drawing up a care plan and providing support after establishment of a diagnosis. This evening was led by an elderly care medicine physician who performed her PhD research on the topic of geriatric needs assessments and integrated care.

The second component comprised of an assessment of cognition and functioning by two study PNs. It started after the baseline measurements were obtained. The PNs were registered nurses trained to administer and interpret cognitive tests, to screen for mood disorders and delirium, and to assess the presence of sensory impairments and whether there was an acute need for additional care. In addition, they were educated about the dementia syndrome and about dementia subtypes. When stage 2 participants had a Mini Mental State Examination (MMSE) score > 1 SD below the (age and education specific) average of healthy persons, or a Visual Association Test (VAT) score ≤ 4, this prompted referral to the FP. The FP would offer further diagnostic evaluation to formally establish or reject a diagnosis of MCI or dementia [47,48]. Our clinical study protocol for the study PNs and FPs was based on the national dementia guideline of the Dutch College of General Practitioners and included criteria for specialist referral [4]. The guideline provides extensive guidance on signaling of dementia, the diagnostic work-up (i.e. history and proxy-history, physical (i.e. neurological) examination, cognitive testing, laboratory testing), establishment of the diagnosis, the differential diagnosis and conditions to exclude (e.g. depression, delirium), criteria for diagnostic referral (e.g. age < 65 years, recent head injury or malignancy) and pharmacological and non-pharmacological management (including care needs assessment, psychosocial interventions for the patient and caregiver) after diagnosis.’ In accordance with the guideline, the final decision to refer was left to the discretion of the FP. Further management of persons diagnosed with MCI and having unmet care needs, or persons diagnosed with dementia, included psychoeducation, a needs assessment, drawing up of a care plan and referral to services. More details of the intervention are provided elsewhere [46].

### Control condition

The control condition consisted of care as usual [49,50]. The standard vocational training of Dutch FPs does not contain education on diagnosing dementia. However, CME is available for all FPs who wish to improve their knowledge or skills on this subject. FPs are expected to know the dementia guideline and adhere to it. Nevertheless, a Dutch study showed that cognitive testing was only performed in 30% of patients in whom dementia was suspected and that adherence to other diagnostic recommendations was also limited [44]. In addition, attitudes regarding diagnosis and disclosure may be rather restrained and knowledge and confidence in their own skills to diagnose limited [44]. International studies also suggest that FPs face many barriers and that their (diagnostic) guideline adherence is limited [51–54].

### Outcomes

Primary outcome (assessed among all eligible patients and among the subgroup participating in stage 2 of the trial): the combined incidence of MCI and dementia diagnoses after 12 months [55]. For the definition of dementia, FPs were referred to the dementia guideline of the Dutch College of General Practitioners (third revision) that adheres to the DSM IV definition of dementia requiring the presence of a memory deficit and impairment in at least one other cognitive domain (e.g. agnosia, executive functioning) resulting in functional impairment, and the absence of a delirium [4,56]. For the definition of MCI they were provided with the definition of Petersen et al. requiring the presence of a memory complaint, memory impairment for age, preserved general cognitive function and activities of daily living and no dementia [57]. We piloted a set of dementia care quality indicators as primary outcome but rejected them because the inter-rater reliability was insufficient.

Secondary outcomes (assessed at baseline, 6 and 12 months among persons participating in stage 2 of the trial and close relatives, if present): mental health and quality of life. Patient mental health was measured by the mental health part of the SF-36; the MH5. Quality of life with the Quality of life in Alzheimer’s Disease scale (QoL-AD) (REF). Close-relatives’ mental health was measured with the General Health Questionnaire (GHQ12),their generic health related Quality of life with the EuroQol scale (EQ5D) [46].

To estimate the diagnostic accuracy of the PNs’ assessments and FPs’ diagnoses at 12 months, we compared these to CAMCOG (cognitive reference test) scores [58]. We used established age and education specific cut-offs for optimal test accuracy. The cut-off for dementia for individuals younger than 75 years of age with low education is < 83; with moderate or high education < 84; for individuals of 75 years of age or older with low education < 65; with moderate or high education < 78 [59]. The cut-off for MCI for individuals with low education is < 26; with moderate or high education < 28 [60]. S1 Fig provides a graphic overview of the trial.

### Sample size calculation

We powered the study to allow detection of a threefold increase of the diagnostic rate based on tripling of the diagnostic rate in a study by Perry et al. in a similar Dutch setting [44,46]. We used a z-test for testing for a difference in proportions. Assuming a power of 70% and an alpha of 0.05, the required sample size was 49 individuals per arm. Assuming an average cluster size of 10 and an intra-class correlation of 0.05 the design effect would equal 1.45 increasing the required sample to 72 per arm. The planned sample size of 162 allowed for 10% loss-to-follow up [46].

### Statistical analysis

The main sample consisted of all persons in whom FPs suspected cognitive impairment at baseline, but without a firm diagnosis of dementia. The subsample comprised persons who agreed to participate in stage 2 of the trial. The primary outcome (difference in incident dementia and MCI diagnoses) was assessed using Generalized Estimating Equations (GEE) analysis. In GEE analyses we used a logistic model with intervention group as the only independent variable. Clustering within practices was taken into account using an exchangeable correlation matrix. Secondary outcomes were analyzed using mixed models. Intracluster correlation coefficients (ICCs) were calculated using Stata®. To assess attrition bias, characteristics of participants who dropped out after baseline were assessed using logistic regression [46].

### Process analysis

We used multiple sources of information to get insight into the fidelity, barriers and facilitators of the intervention for care providers and patients: the qualitative data concerned written observations of the PNs; meeting notes and approved verbatim transcripts of semi-structured interviews with FPs (n = 5). Due to limited time and resources and the challenges posed by triangulation of all these sources the analysis of the qualitative data was done in an iterative rather than systematic way. The quantitative data concerned the results of a self-developed brief questionnaire for stage 2 participants, addressing whether they recalled the consultation with the PN, whether they considered the test as useful and whether the results were explained to them [61].

## Results

### Recruitment and participant flow

Fig 1 provides a diagram of participant flow. Patients were included between October 4, 2011 and May 31, 2012; the follow-up ran untill August 3, 2013. The primary outcome was obtained for all persons from the main sample (n = 647). A total of 145 persons participated in stage 2 of the trial. Drop-outs (n = 21/145) of stage 2 had a significantly worse QoL-AD score (33.1 [SD 5.1] versus 37.4 [SD 4.8]; p = 0.001), and lower MMSE score (22.9 [SD 3.3] versus 25.4 [SD 3.8]; p = 0.005), at baseline.

### Baseline characteristics

Table 1 provides an overview of baseline characteristics of PCPs, older persons with suspected cognitive impairment and relatives.

**Table 1:** Baseline characteristics.

	Control	Intervention	
**Primary care practices**	N = 8		N = 7
Patients 65+/all patients (%)	4,099/24,746 (16.6)		3,837/23,269 (16.5)
Geriatric Nurse present, n (%)	5 (62.5)		5 (71.4)
**Participants trial stage 1**	n = 321		n = 326
Age, mean (SD)	79.8 (7.1)		80.3 (7.5)
Sex male, n (%)	127 (39.6)		145 (44.5)
**Participants trial stage 2**	n = 63		n = 82
Age, mean (SD)	78.6 (6,4)		77.8 (6,6)
Sex male, n (%)	39 (61.9)		45 (54.9)
Contacts FP previous 6 months, mean (SD)	2.3 (1.7)		2.9 (3.7)
Mental health (MH5), mean (SD)	72.6 (18.2)		71.0 (19.5)
Quality of life (EQ5D), mean (SD)	0.75 (0.20)		0,78 (0.22)
Quality of life (QoL-AD), mean (SD)	36.7 (5.6)		36.9 (4.7)
Number of comorbidities, mean (SD)	2.5 (1.9)		2.1 (1.8)
Behavioral symptoms (NPI), mean (SD)	8.0 (11.4)		6.4 (7.5)
Presence of close relative, n (%)	54 (85.7)		68 (82.9)
Presence of chronic disease, n (%)	29 (58.0)		29 (43.9)
Presence of psychiatric disease, n (%)	10 (20.0)		10 (15.2)
ADL and iADL problems (Katz15), mean (SD)	3.3 (2.6)		1.8 (2.6)
Presence of dementia (CAMCOG), n (%)	20 (33.3)		25 (31.6)
Presence of dementia/MCI (CAMCOG), n (%)	26 (42.6)		33 (41.8)
Memory problems (MMSE), mean (SD)	25.3 (3.0)		24.8 (3.8)
**Close relative**	n = 54		n = 68
Sex male, n (%)	18 (36.0)		23 (35.4)
Mental health (GHQ12), mean (SD)	1.54 (2.42)		1.62 (2.66)
Quality of life (EQ5D), mean (SD)	0.82 (0.22)		0.86 (0.11)
Sense of competence to provide care (SSCQ),mean (SD)	29.4 (4.9)		30.2 (4.7)
Social support (SSL12), mean (SD)	27.3 (5.5)		29.0 (5.8)

SD = standard deviation, MH5 = Mental Health part of SF-36, EQ5D = EuroQol, QoL-AD = Quality of life in Alzheimer’s Disease scale, NPI = Neuropsychiatric Inventory, Katz15 = 15 item Katz questionnaire, CAMCOG = Cambridge Cognitive Examination, MMSE = Mini Mental State Examination, GHQ12 = 12-item General Health Questionnaire, SSCQ = Short Sense of Competence Questionnaire, SSL12 = 12-item Social Support List.

### Primary outcome: New MCI and dementia diagnoses after 12 months

Intention to treat analysis of new diagnosis of MCI or dementia among all persons from the main sample (n = 647; Table 2: New diagnoses among stage 1 participants) yielded 42.3% (138/326) new diagnoses in the intervention and 30.5% (98/321) in the control group. The difference was not significant when GEE was applied to account for clustering (estimated difference: 10.8%, OR: 1.55, 95% confidence Interval 0.62–3.89).

**Table 2:** New diagnoses by family physicians at 12-months follow-up.

**New diagnoses among stage 1 participants**	**Control** (n = 321)	**Intervention** (n = 326)
Dementia, n (%)	38 (11.8%)	40 (12.3%)
Mild Cognitive Impairment, n (%)	60 (18.7%)	98 (30.1%)
Other cognitive impairment (e.g. traumatic, stroke) n (%)	13 (4.0%)	13 (4.0%)
Normal for age, n (%)	169 (52.6%)	133 (40.8%)
FPs could not label, n (%)	41 (12.8%)	42 (12.9%)
**New diagnoses among stage 2 participants**	**Control** (n = 63)	**Intervention** (n = 82)
Dementia, n (%)	6 (9.5%)	7 (8.5%)
Mild Cognitive Impairment, n (%)	19 (30.2%)	27 (32.9%)
Other cognitive impairment (e.g. traumatic, stroke) n (%)	4 (6.3%)	5 (6.1%)
Normal for age, n (%)	30 (47.6%)	40 (48.8%)
FPs could not label, n (%)	4 (6.3%)	3 (3.7%)

Among persons participating in stage 2 of the study (n = 145; Table 2: New diagnoses among stage 2 participants), the difference in new diagnoses was marginal: 41.5% (34/82) new diagnoses in the intervention and 39.7% (25/63) in the control group (estimated difference adjusted for ADL and iADL dependency: 3.3%, OR: 1.12, 95% Confidence Interval 0.47–2.74). Since there was no significant difference we did not perform the planned moderation analysis.

### Secondary outcomes

There were no significant differences in mental health and quality of life between stage 2 participants from the intervention and control group or their relatives (Table 3).

**Table 3:** Mental health and quality of life of stage 2 participants at follow-up.

Outcome	Control	Intervention	ICC	Time x group	
	n = 57 n-r = 50	n = 67 n-r = 54		F	Sig.
*Mental health elderly (MH5)(range 0–100)*			<0.001	.016	.899
Baseline, mean (SD)	72.63 (18.18)	71.04 (19.47)			
6 months, mean (SD)	77.67 (14.13)	75.56 (16.69)			
12 months, mean (SD)	71.67 (18.76)	71.46 (18.37)			
*Mental health close relative (GHQ12)(range 0–12)*			<0.001	.179	.673
Baseline, mean (SD)	1.54 (2.42)	1.62 (2.66)			
6 months, mean (SD)	1.30 (2.20)	1.60 (2.45)			
12 months, mean (SD)	1.39 (2.10)	1.72 (2.92)			
*Quality of life elderly (EQ5D)(range -0*.*33–1)*			<0.001	.038	.847
Baseline, mean (SD)	0.75 (0.20)	0.78 (0.22)			
6 months, mean (SD)	0.77 (0.22)	0.79 (0.20)			
12 months, mean (SD)	0.75 (0.20)	0.78 (0.21)			
*Quality of life elderly (QoL-AD)(range 13–52)*			<0.001	.502	.481
Baseline, mean (SD)	36.69 (5.56)	36.92 (4.72)			
6 months, mean (SD)	36.75 (4.79)	37.36 (4.84)			
12 months, mean (SD)	35.59 (4.58)	36.44 (4.53)			
*Quality of life close relative (EQ5D)(range -0*.*33–1)*			.024	1.223	.272
Baseline, mean (SD)	0.82 (0.22)	0.86 (0.11)			
6 months, mean (SD)	0.86 (0.16)	0.87 (0.14)			
12 months, mean (SD)	0.85 (0.16)	0.88 (0.13)			
*sense of competence to provide care*, *close relative (SSCQ) (range 0–35)*			<0.001	.109	.742
Baseline, mean (SD)	29.37 (4.85)	30.23 (4.73)			
6 months, mean (SD)	29.35 (4.31)	30.23 (4.54)	** **	** **	** **
12 months, mean (SD)	28.90 (4.31)	29.69 (4.52)	** **	** **	** **

F = F-test for time x group, Sig. = p value for time x group, ICC = intracluster correlation coefficient, SD = standard deviation, n = number of patients, n-r = number of relatives, MH5 = Mental Health part of SF-36, EQ5D = EuroQol, QoL-AD = Quality of life in Alzheimer’s Disease scale, MMSE = Mini Mental State Examination, GHQ12 = 12-item General Health Questionnaire, SSCQ = Short Sense of Competence Questionnaire.

#### Agreement between results of case finding and the CAMCOG

The assessments by PNs identified only five of 21 persons with cognitive impairment according to the CAMCOG, with no false positives. When FP diagnostic labels of MCI/dementia as listed at 12 months (after both components of the intervention were finished) were compared with CAMCOG scores at 12 months, 6/ 19 (31.6%; 2 MCI, 4 dementia) diagnoses were missed in intervention and 12/ 24 (50%; 6 MCI, 6 dementia) in control practices. In addition, the comparison yielded 15/44 (34.1%; 13 MCI, 2 dementia) false positives in intervention and 10/24 (41.7%; 9 MCI, 1 dementia) in control practices.

### Process evaluation

#### Participation and intervention uptake

All FPs in the intervention group participated in the post-graduate training. However, only 25% (145/578) of persons eligible to stage 2 of the trial consented to participate (Fig 1). Of stage 2 participants in the intervention group, 30% (25/82) were not assessed by the PN. Reasons included: not possible to reach; drop-out; deterioration of clinical condition; refusing to participate on reconsideration.

#### Adherence to the clinical study protocol

The PNs reported to the FPs about everyone they assessed and composed a written summary. This included test results, a conclusion about the cognitive status and presence of sensory or functional impairment and whether there was a need for additional care. FPs did not always follow the recommendations of the clinical protocol (overview in S2 Fig) and instead applied watchful waiting. Because of the low MCI and dementia incidence, PNs performed very few geriatric assessments (RAI) [62].

#### Older persons’ experiences

The experiences of stage 2 participants were explored by a brief questionnaire. The minority of participants who remembered the assessment (12/57) experienced the test as useful and remembered that the results were explained to them.

#### Family physicians’ experiences

FPs from the intervention group considered the training useful. The intervention helped them be more pro-active and to support the informal caregiver sooner (e.g. arranging day-care for the patient). They thought it might help to keep patients at home longer. The periodic cognitive assessments allowed observing a trend in time. They considered it reassuring to know that certain people did not have cognitive deficits. FPs had the impression that the intervention was well received by their patients.

#### Practice nurses’ experiences

Inviting older people for the assessment appeared time-consuming. On several occasions the PNs suspected cognitive decline in people who scored above the cut-offs on the cognitive screening tests. The PNs observed that, while study participants welcomed full disclosure about the results of the cognitive assessment, some non-participants would only want to know in case ‘all is well’.

## Discussion

### Main findings

After one year, more new diagnoses of MCI were found in the intervention group compared to the control group, but this difference was not statistically significant after adjustment for clustering. There was no difference in the number of new dementia diagnoses between the intervention and control group. Only a fourth of eligible patients were willing to participate in the second part of the trial. In this group, no beneficial or harmful impact of the intervention was found.

### Limitations

The low response rate limits the external validity of stage 2 outcomes of the trial. Although a non-response analysis did not show selective (non-)response, we cannot rule it out [63]. The prior chance of MCI and dementia appeared lower than estimated designing the study [46]. In addition, the contrast may have been decreased by a Hawthorne effect in the control group, or by the keen interest in dementia care of some FPs in control practices also providing nurse-led elderly care. Regarding the intervention, the second component of the intervention did not reach all stage 2 participants. Furthermore, the sensitivity of the cognitive tests performed by the PNs at the applied cut-offs may have been too low: the prevalence of possible dementia based on CAMCOG scores was four times higher than that based on the PNs’ assessments. Also, FPs did not perform further diagnostic assessment in all persons referred by the PN. This may be due to following a strategy of watchful waiting (an effective diagnostic strategy in low prevalence settings) or to barriers like lack of time, the complexity of establishing a dementia diagnosis or to the lack of a diagnostic demand by the patient or caregiver[44,53,64]. These factors may have diluted the potential impact of the intervention on the rate of new diagnosis. Regarding the use of the CAMCOG: this instrument is not considered as gold standard for the diagnosis of dementia. However, it is a fairly reliable measure for the presence (or absence) of dementia and the memory section can predict prodromal Alzheimer in MCI patients fairly accurate [59,65].

### Interpretation

We assume that the main reasons for the absent effects of the intervention were the diagnostic efforts in the control practices, the lower than estimated prior chance (and severity) of cognitive impairment in the sample and the limited adherence of FPs to the study protocol. The absence of a difference in the number of new diagnoses between groups suggests that insufficient knowledge of MCI and dementia diagnosis of FPs was no substantial barrier to diagnosis in the studied practices. However, we cannot draw final conclusions about this, since we did not test FPs’ knowledge before and after the training and because we cannot fully rule out a type II error. Only a minority of invited people participated in stage 2 of the trial. The reasons for this (most often lack of interest or too burdensome) do not give insight into whether people also avoided it because of the cognitive assessment. That we found no effect on mental functioning is most likely attributable to the absent effect on new diagnoses and possibly the relatively low overall burden of (cognitive) disease [32].

### Our findings in the context of previous research

Two earlier RCTs reported a positive effect of an educational intervention on a) the number of dementia diagnoses and b) the ability to establish this diagnosis without specialist support [43,44]. Either the patient, family or the FP may have experienced a higher need for diagnostic evaluation than in our study and the prior chance of dementia was likely higher [43,44]. In addition, in our study there was no collaboration with a specialist, which may have stimulated a more proactive approach in other studies [41,44,66]. Two other studies aimed at less selected populations found no effect on guideline adherence or the number of new diagnoses [67,68].

### Implications for practice

At present we cannot recommend case finding of MCI and dementia among persons in whom FPs consider cognitive impairment or dementia ‘possibly present’. Nevertheless, clinicians may want to focus on improving the diagnostic procedure for this group. Based on the absence of a negative impact of our intervention on mental health, and on the favorable experiences of the FPs and PNs, we would recommend not to be overly careful to discuss and to assess whether or not older persons have cognitive deficits. Based on the comparison of the CAMCOG results with the PNs’ screening results, presently used cognitive tests on their own appear insufficient for the initial detection of (earlier stages of) cognitive decline; we would recommend that initial assessments include a careful history, proxy history and an assessment of functioning [69].

### Implications for future research

Future case finding studies may target persons with a higher prior chance of dementia by combining FPs clinical judgment with clinical predictors of dementia (e.g. high age, vascular risk factors), and with predictors of missed dementia diagnoses (e.g. living alone, low contact frequency) [41,70,71]. Collaboration with PNs and low-threshold consultation of dementia specialists also deserve further exploration [44,66]. Finally, FPs need better tools to distinguish between normal ageing and dementia, and possibly to distinguish between normal ageing and MCI with a high risk of conversion to dementia (i.e. multi-domain MCI) [72,73].

## Conclusion

We did not find a significant increase in MCI and dementia diagnoses resulting from a combined educational, case finding and care intervention. Case finding did not seem to have impact on persons’ mental health. Because of the possibility of a type II error and the suboptimal implementation of the intervention, we cannot rule out potential clinical effectiveness of the intervention. Therefore, a larger study with more focus on barriers to implementation is warranted. In addition, we would recommend that future case finding studies target groups with a higher probability of dementia, for example by using EMR derivable risk factors for dementia and/or for a missed diagnosis of dementia.

## Supporting Information

S1 CONSORT Checklist (DOCX)

S1 Fig Overview of the trial. PN = Practice nurse, FP = family physician, * see S2 Fig ‘overview of component 2 of the intervention’ for indications for assessment the FP and for further care.
(DOCX)

S2 Fig Overview of component 2 of the intervention. PN = practice nurse, MMSE = Mini Mental State Examination, VAT = Visual Association Test, RAI-CA = Resident Assessment Instrument–Contact Assessment to assess short term need for additional services, prime-MD = Primary Care Evaluation of Mental Disorders screening questionnaire for depressive symptoms, DCGP = Dutch College of General Practitioners, FP = family physician, RAI Home care = comprehensive geriatric assessment, MCI = Mild Cognitive Impairment.
(DOC)

S1 File Brief instruction with CRF. (DOC)

S1 Protocol (DOC)

S1 Table CRF primary outcome (XLS)

## Abbreviations

ADL: Activities of daily living
iADL: Instrumental activities of daily living
CAMCOG: Cambridge cognitive examination
EQ5D: The 5-item EurQol questionnaire
FP: Family physician
GEE: Generalized estimating equations
GHQ12: The 12-item general health questionnaire
ICC: Intracluster correlation coefficient
MCI: Mild cognitive impairment
MH5: The 5-item mental health questionnaire (part of the SF-36
MMSE: Mini mental state examination
PCP: Primary care practice
PN: Practice nurse
QoL-AD: Quality of life in Alzheimer’s Disease questionnaire
RAI: Resident assessment instrument
RCT: Randomized clinical trial
VAT: Visual association test
