# Supplementary material for: Case Finding of Mild Cognitive Impairment and Dementia and Subsequent Care; Results of a Cluster RCT in Primary Care
Source: PLoS One. 2016 Jun 16;11(6):e0156958. doi: 10.1371/journal.pone.0156958 (PMC4910994; doi:10.1371/journal.pone.0156958)
Supplement: S1 Fig — PN = Practice nurse, FP = family physician, * see S2 Fig ‘overview of component 2 of the intervention’ for indications for assessment the FP and for further care. (DOCX) [file pone.0156958.s002.docx]

**S1 Fig. Overview of the trial**

PN = Practice nurse, FP = family physician, * see S2 Fig ‘overview of component 2 of the intervention’ for indications for assessment the FP and for further care

Secondary outcome (12 months):

mental health older person & relative

- No assessment by study PN

- Diagnosis and care as usual

- Assessment PN

- When indicated assessment FP*

- When indicated referral, information, support and care*

Participants stage 2

(n = 82)

Participants stage 2

(n = 63)

FPs not trained

FPs trained to diagnose MCI and dementia

Primary outcome (12 months):

new MCI and dementia diagnoses

Persons labeled ‘possible cognitive impairment or dementia’ by FPs

(n = 321)

Persons labeled ‘possible cognitive impairment or dementia’ by FPs

(n = 326)

**Control**

**Intervention**
