# Supplementary material for: Case Finding of Mild Cognitive Impairment and Dementia and Subsequent Care; Results of a Cluster RCT in Primary Care
Source: PLoS One. 2016 Jun 16;11(6):e0156958. doi: 10.1371/journal.pone.0156958 (PMC4910994; doi:10.1371/journal.pone.0156958)
Supplement: S2 Fig — PN = practice nurse, MMSE = Mini Mental State Examination, VAT = Visual Association Test, RAI-CA = Resident Assessment Instrument–Contact Assessment to assess short term need for additional services, prime-MD = Primary Care Evaluation of Mental Disorders screening questionnaire for depressive symptoms, DCGP = Dutch College of General Practitioners, FP = family physician, RAI Home care = comprehensive geriatric assessment, MCI = Mild Cognitive Impairment. (DOC) [file pone.0156958.s003.doc]

Appendix 2. Overview of component 2 of the intervention

**Possible cognitive impairment**

Screening PN

Consultation FP

MMSE > 1SD below mean / VAT ≤ 4

MMSE, VAT, RAI-CA, prime-MD, hearing, vision.

RAI Contact Assessment

b) Physical & neurological examination

c) Medication review

- Psychoeducation and support

- RAI Home Care if dementia or if MCI ánd positive RAI Contact Assessment

- Care plan

- Referral to services (e.g. home care)

**Yes**

**Revision care plan every 6 months or earlier if required**

**Diagnosis of dementia or MCI?**

**Yes**

**No**

DCGP-guideline recommendations:

a) Laboratory tests

Assessment and care PN

**Revision screening PN every 6 months**

c) Disclosure diagnosis of dementia

d) Diagnostic referral?

PN = practice nurse, MMSE = Mini Mental State Examination, VAT = Visual Association Test, RAI-CA = Resident Assessment Instrument – Contact Assessment to assess short term need for additional services, prime-MD = Primary Care Evaluation of Mental Disorders screening questionnaire for depressive symptoms, DCGP = Dutch College of General Practitioners, FP = family physician, RAI Home care = comprehensive geriatric assessment, MCI = Mild Cognitive Impairment
