# Supplementary material for: Case Finding of Mild Cognitive Impairment and Dementia and Subsequent Care; Results of a Cluster RCT in Primary Care
Source: PLoS One. 2016 Jun 16;11(6):e0156958. doi: 10.1371/journal.pone.0156958 (PMC4910994; doi:10.1371/journal.pone.0156958)
Supplement: S1 File — (DOC) [file pone.0156958.s004.doc]

Planning and instruction

#### If possible we would like to recollect the USB-stick on - *date* - at your practice. We will send you a reminder in one week.

We request you to reserve the category **dementia syndrome** for those persons in whom you or a colleague established this diagnosis.

When there is uncertainty about the diagnosis we request you not to classify as dementia syndrome.

Consider using the classification ‘mild cognitive impairment’.

Below a short description of the category **mild cognitive impairment:**

This classification is used when cognitive decline is evident but when it does not result in functional impairment.

We wish you success filling out the form!

With kind regards,

Pim van den Dungen on behalf of the COMPAS team
