# Supplementary material for: Case Finding of Mild Cognitive Impairment and Dementia and Subsequent Care; Results of a Cluster RCT in Primary Care
Source: PLoS One. 2016 Jun 16;11(6):e0156958. doi: 10.1371/journal.pone.0156958 (PMC4910994; doi:10.1371/journal.pone.0156958)
Supplement: S1 Protocol — (DOC) [file pone.0156958.s005.doc]

**PROTOCOL TITLE**

**COMPAS-D**

**Elderly patients suspected of cognitive decline by family physicians; a cluster randomized controlled trial to improve the diagnostic and care process**

| **Protocol ID** | **WC2011-040** |
| --- | --- |
| **Short title** | **COMPAS-D** |
| **Version** | **3** |
| **Date** | **15-7-2011** |
| **Coordinating investigator/project leader** | **Dr. H.P.J. van Hout, Huisartsgeneeskunde, EMGO+, VUmc, hpj.vanhout**[**@vumc.nl**](mailto:g.nijpels@vumc.nl) |
| **Principal investigator(s) (in Dutch: hoofdonderzoeker/uitvoerder)**  ***Multicenter research: per site*** | **Drs. P. van den Dungen, Huisartsgeneeskunde, EMGO+, VUmc, p.vandendungen**[**@vumc.nl**](mailto:g.nijpels@vumc.nl) |
|  |  |
| **Sponsor (in Dutch: verrichter/opdrachtgever)** | **Stg. Stoffels & ZonMw** |
|  |  |
| **Independent physician(s)** | **Dr. A.H. Blankenstein**  **Afdeling huisartsgeneeskunde, EMGO+**  **Van der Boechorststraat 7**  **1081 BT AMSTERDAM**  **020-4448198** |
| **Laboratory sites <*if applicable*>** | ***Not applicable*** |
| **Pharmacy <*if applicable*>** | ***Not applicable*** |
|  |  |

AANPASSING NAV LOSSE EINDJES 15 JULI 2011 28 nov 2011 (gehoor, visus)

**PROTOCOL SIGNATURE SHEET**

| **Name** | **Signature** | **Date** |
| --- | --- | --- |
| **Sponsor or legal representative:**  ***<please include name and function>***  **For non-commercial research,**  **Head of Department:**  ***<include name and function>*** | ***Niet van toepassing, non-commercial research***  **Prof. Dr. HE van der Horst**  **Hoofd afdeling huisartsgeneeskunde, EMGO+, VUmc** |  |
| **Coordinating Investigator/Project leader/Principal Investigator:**  ***<please include name and function>*** | **Dr. H.P.J van Hout**  **Assistant-professor, afdeling huisartsgeneeskunde, EMGO+, VUmc** |  |
|  |  |  |

**TABLE OF CONTENTS**

[SUMMARY 6](#__RefHeading___Toc291573812)

[1. INTRODUCTION AND RATIONALE 8](#__RefHeading___Toc291573813)

[2. OBJECTIVES 9](#__RefHeading___Toc291573814)

[3. STUDY DESIGN 10](#__RefHeading___Toc291573815)

[*A flow chart can be included to give a overview of the procedures that subjects will undergo in the course of research.>* 10](#__RefHeading___Toc291573816)

[STUDY POPULATION 11](#__RefHeading___Toc291573817)

[4.1 Population (base) 11](#__RefHeading___Toc291573818)

[4.2 Inclusion criteria 11](#__RefHeading___Toc291573819)

[4.3 Exclusion criteria 11](#__RefHeading___Toc291573820)

[4.4 Sample size calculation 11](#__RefHeading___Toc291573821)

[5. TREATMENT OF SUBJECTS 12](#__RefHeading___Toc291573822)

[5.1 Investigational product/treatment 12](#__RefHeading___Toc291573823)

[5.2 Use of co-intervention (if applicable) not applicable 14](#__RefHeading___Toc291573824)

[5.3 Escape medication (if applicable) not applicable 14](#__RefHeading___Toc291573825)

[5.3 15](#__RefHeading___Toc291573826)

[6 INVESTIGATIONAL MEDICINAL PRODUCT 15](#__RefHeading___Toc291573827)

[7. METHODS 15](#__RefHeading___Toc291573828)

[7.1 Study parameters/endpoints 15](#__RefHeading___Toc291573829)

[7.2 Randomisation, blinding and treatment allocation 16](#__RefHeading___Toc291573830)

[7.3 Study procedures 16](#__RefHeading___Toc291573831)

[7.4 Withdrawal of individual subjects 20](#__RefHeading___Toc291573832)

[7.5 Replacement of individual subjects after withdrawal 20](#__RefHeading___Toc291573833)

[7.6 Follow-up of subjects withdrawn from treatment 20](#__RefHeading___Toc291573834)

[7.7 Premature termination of the study 20](#__RefHeading___Toc291573835)

[8. SAFETY REPORTING 21](#__RefHeading___Toc291573836)

[8.1 Section 10 WMO event 21](#__RefHeading___Toc291573837)

[8.2 Adverse and serious adverse events 21](#__RefHeading___Toc291573838)

[8.3 Follow-up of adverse events 23](#__RefHeading___Toc291573839)

[8.4 Data Safety Monitoring Board (DSMB) 23](#__RefHeading___Toc291573840)

[9. STATISTICAL ANALYSIS 24](#__RefHeading___Toc291573841)

[9.1 Descriptive statistics 24](#__RefHeading___Toc291573842)

[9.2 Univariate analysis 24](#__RefHeading___Toc291573843)

[9.3 Multivariate analysis 24](#__RefHeading___Toc291573844)

[9.4 Interim analysis (if applicable) 24](#__RefHeading___Toc291573845)

[10. ETHICAL CONSIDERATIONS 25](#__RefHeading___Toc291573846)

[10.1 Regulation statement 25](#__RefHeading___Toc291573847)

[10.2 Recruitment and consent 25](#__RefHeading___Toc291573848)

[10.3 Objection by minors or incapacitated subjects (if applicable) 25](#__RefHeading___Toc291573849)

[10.4 Benefits and risks assessment, group relatedness 25](#__RefHeading___Toc291573850)

[10.5 Compensation for injury 25](#__RefHeading___Toc291573851)

[10.6 Incentives (if applicable) 26](#__RefHeading___Toc291573852)

[11. ADMINISTRATIVE ASPECTS AND PUBLICATION 27](#__RefHeading___Toc291573853)

[11.1 Handling and storage of data and documents 27](#__RefHeading___Toc291573854)

[11.2 Amendments 27](#__RefHeading___Toc291573855)

[11.3 Annual progress report 27](#__RefHeading___Toc291573856)

[11.4 End of study report 27](#__RefHeading___Toc291573857)

[11.5 Public disclosure and publication policy 28](#__RefHeading___Toc291573858)

[12. Appendices 28](#__RefHeading___Toc291573859)

[13. REFERENCES 33](#__RefHeading___Toc291573860)

**LIST OF ABBREVIATIONS AND RELEVANT DEFINITIONS**

| **ABR** | **ABR form (General Assessment and Registration form) is the application form that is required for submission to the accredited Ethics Committee (ABR = Algemene Beoordeling en Registratie)** |
| --- | --- |
| **AE** | **Adverse Event** |
| **AR** | **Adverse Reaction** |
| **CA** | **Competent Authority** |
| **CCMO** | **Central Committee on Research Involving Human Subjects** |
| **CV** | **Curriculum Vitae** |
| **DSMB** | **Data Safety Monitoring Board** |
| **EU** | **European Union** |
| **EudraCT** | **European drug regulatory affairs Clinical Trials GCP Good Clinical Practice** |
| **IB** | **Investigator’s Brochure** |
| **IC** | **Informed Consent** |
| **IMP** | **Investigational Medicinal Product** |
| **IMPD** | **Investigational Medicinal Product Dossier** |
| **METC** | **Medical research ethics committee (MREC); in Dutch: medisch ethische toetsing commissie (METC)** |
| **(S)AE** | **Serious Adverse Event** |
| **SPC** | **Summary of Product Characteristics (in Dutch: officiële productinfomatie IB1-tekst)** |
| **Sponsor** | **The sponsor is the party that commissions the organisation or performance of the research, for example a pharmaceutical**  **company, academic hospital, scientific organisation or investigator. A party that provides funding for a study but does not commission it is not regarded as the sponsor, but referred to as a subsidising party.** |
| **SUSAR** | **Suspected Unexpected Serious Adverse Reaction** |
| **Wbp** | **Personal Data Protection Act (in Dutch: Wet Bescherming Persoonsgevens)** |
| **WMO** | **Medical Research Involving Human Subjects Act (Wet Medisch-wetenschappelijk Onderzoek met Mensen** |

# SUMMARY

**Rationale:** In sight of the upcoming dementia epidemic it is of vital importance to diagnose dementia accurately and early and to optimize care for home dwelling dementia patients and their informal caregivers. At present, diagnosis and management of dementia in primary care are suboptimal. Previous studies suggest that the combination of training providers, deploying a care coordinator and structuring collaboration among care providing organisations could improve the recognition of dementia; the quality of subsequent care for home dwelling dementia patients and their wellbeing. Cost effectiveness was not yet assessed.

**Research questions:**

1) To what extent does pro-active case finding of Mild Cognitive Impairment (MCI) and dementia increase the number of diagnoses in patients suspected of cognitive impairment by family physicians (FPs)?

2) To what extent does case finding and subsequent collaborative care improves the quality of care in patients diagnosed with mild cognitive impairment (MCI) or dementia diagnoses?

3) Do case finding and subsequent collaborative care improve the wellbeing, meet the information needs and decrease worrying of patients diagnosed with dementia or mild cognitive impairment (MCI) and their relatives?

4) What is the accuracy of FPs’ classification of the presence and absence of cognitive impairment in their older patients ≥ 65 years?

**Setting and Study population:**

The cluster RCT is executed in 16 primary care practices (PCPs) among 312 patients ≥ 65 years in whom FPs suspect cognitive impairment.

The diagnostic accuracy study is executed among 312 patients labelled ‘possible cognitive impairment’ and in 100 patients labelled ‘no signs of cognitive impairment’ by FPs.

**Study design:**

Ad 1-3) Cluster Randomised Controlled Trial.

Intervention: collaborative diagnostic evaluation and management.

A trained practice nurse invites patients with suspected cognitive impairment for a comprehensive geriatric assessment, including a cognitive screening. If this assessment suggests the presence of MCI or dementia, it is followed by a more thorough assessment, including physical and neurological examination and laboratory tests, by the FP. When this results in a MCI or dementia diagnosis, the practice nurse in close collaboration with the FP will offer patients collaborative care. This consists of psycho education and support. In addition the practice nurse according to their needs as identified will coordinate care after a geriatric assessment.

Controls: Care as usual will be offered by the FP.

Ad 4) Diagnostic Test Accuracy Study: cross-sectional comparison of FPs’ recollections of the presence or absence of cognitive impairment in their patients ≥ 65 years, with reference standard diagnoses on the presence or absence of MCI or dementia.

**Main study parameters**

Ad 1-2 (b) 23 quality indicators: 13 on the diagnostic process, 3 on follow-up care, 4 on structure of care and 3 on outcomes after 12 months.

(a) The number of MCI and dementia diagnosis after 12 months.

Ad 3. Patient: Quality of life (EQ5D, QoL-AD); mental wellbeing (MH5); worrying (…), information needs (…). Caregiver: mental health (GHQ 12); burden (SSCQ); quality of life (EQ-5D); worrying (..), information needs (…) over 12 months.

Ad 4. Diagnostic accuracy of FPs’ cognitive classification of individuals compared to the reference standard (CAMCOG) diagnosis at baseline.

**Nature and extent of the burden and risks associated with participation, benefit and group relatedness:** The interventions done within the context of the study are regarded to be of no risk to the patients and caregivers exposed to them. We aimed to minimise the burden placed on dementia patients and their caregivers by limiting the interviews with patients and carers (and questionnaires for carers) to twice a year.

# 1. INTRODUCTION AND RATIONALE

Dementia is a condition that is characterised by progressive dependency. In the advanced stages dementia demands either professional or informal care or a combination of these two. Worldwide dementia prevalence is predicted to rise from 35.6 million in 2010 to 65.7 million in 2030.(Ferri et al. 2009)

In most developed countries dementia care is already at the top of the list of conditions weighing heaviest on health care expenditures and growing fastest.

In gate keeping primary health care systems like the Netherlands, FPs play a pivotal role in the recognition and diagnosis of dementia and in linking or referring patients and caregivers to secondary dementia care (such as memory clinics and case management programs) and other community services. Although both dementia patients and informal caregivers are likely to benefit from earlier recognition and diagnosis of the condition(Boise et al. 2004;Sandholzer et al. 1999), dementia is still often diagnosed late in the disease process(Boise et al. 2004;Bradford et al. 2009;Meerman et al. 2008). Educational interventions aimed at FPs have the potential to raise the rate of diagnosis(Downs 2006). Additional coaching of FPs and collaboration of FPs with trained health care nurses, specialised in dementia diagnosis and management results in better adherence to diagnostic guidelines and improved rates of disclosure and education of patients and caregivers(Perry et al. ;Perry et al. 2008).

At present, management of dementia patients and their informal carers in primary care is predominantly following demands rather than being provided proactively. Adherence to dementia guidelines is low(Hinton et al. 2007;Wenger et al. 2003) and although FPs consider assessment of care needs of patient and caregiver important, this is often not achieved in daily practice(Schoenmakers et al. 2009;Van Hout et al. 2000). FPs acknowledge that behavioural problems, an important predictor of caregiver burden, are treated suboptimally (Hinton et al. 2007). Nevertheless, they appear to be poorly informed about the content of community support services and only a small proportion of North-American FPs refer to such services(Cody et al. 2002;Schoenmakers et al. 2009;Yaffe et al. 2008).

With the upcoming dementia epidemic, we consider it important to structurally improve management of dementia in primary care, with assessment of care needs of patients and caregivers, composing (and revising) of care plans for both patients and caregivers and referral to community services or regional care providers according to their needs. Previous research has shown that multifaceted interventions, such as education and support of FPs and building strong collaboration amongst dementia care providers and services in a region are potentially effective.

Vickrey et al. showed significant effects on quality of care and caregiver outcomes of such an intervention. They implemented at the primary care level a structured assessment by a casemanager, algorithms linking specific care management actions to assessment results and interorganizational care coordination and referral protocols(Vickrey et al. 2006). Reuben et al also attempted to improve dementia care by means of a multifaceted intervention(Reuben et al. 2003). They failed at their first attempt but learned that physicians perform better at medical tasks then at counselling and education. Furthermore physicians are often unaware of various community services and relatively inexperienced in the treatment of behavioural symptoms. The authors collaborated with FPs to tailor the intervention more to the their specific perceived demands and needs and succeeded to improve some aspects of quality of care and to improve interdisciplinary collaboration(Reuben et al. 2010). Where Perry et al. did show effects of their multifaceted intervention on quality of the diagnostic process, they did not find significant effects on the quality of the follow-up care/long term care process for patient and caregiver(Perry et al. 2008).

FPs indicate they need (and welcome) support to improve care (Cody et al. 2002;Van Hout et al. 2000). Previous research demonstrated that nurses are better at managing care then at diagnosing dementia. Particularly management of behavioural symptoms is perceived to be difficult(Bryans et al. 2003). Callahan showed that collaboration within an interdisciplinary team led by an advanced practice nurse had significant effects on medication usage and on behavioural and psychological symptoms of dementia patients(Callahan et al. 2006).

Adequate management of behavioural and psychological problems is however time consuming and requires specific expertise(Chenoweth et al. 2009). This task may therefore be more suitable to be performed by specialised case managers or mental health workers.

In conclusion: Both the recognition and diagnosis of cognitive impairment and the care for home dwelling persons with dementia and their informal carers need to be improved. Potentially effective strategies have been identified. These interventions were however not effective in all studies. The collaborative team of FP and practice nurse can play an important part in the *diagnosis* of dementia, the *assessment* of needs of persons with dementia and their caregivers and in the *provision* and *coordination* of stepped dementia care. FPs and nurses both have their specific strengths and weaknesses that need to be taken into consideration in the division of tasks. This collaborative model deserves further development and evaluation as it is potentially a high quality, cost-effective complement to secondary care dementia services.

# 2. OBJECTIVES

Our primary objective is to estimate whether a multifaceted intervention including collaboration of FPs and trained practice nurses in the diagnosis of mild cognitive impairment (MCI) and dementia and in subsequent management improve the quality of the diagnostic process and the quality of care.

Secondary objectives are to investigate:

a) Whether this intervention improves the general wellbeing of dementia patients and their informal caregivers;

b) The accuracy of the FPs’ cross-sectional classification of the cognitive status of all patients ≥ 65 years and c) whether the intervention is cost-effective

d) Success and failure factors of this intervention.

# 3. STUDY DESIGN

To estimate the effects of the intervention on quality of the diagnostic process, quality of care and on patient and caregiver wellbeing, a stepped wedged cluster RCT design over 24 months will be used. Stepped wedged means that all practices eventually apply the intervention but the starting point per practice is randomised in early and late starters (>12 months). This design is also used for the cost-effectiveness analysis (Figure 1).

To study the accuracy of the FPs cognitive classification we will use a classical Diagnostic Test Accuracy Design. The cross sectional classification of the FP is compared to a reference standard diagnosis. Both patients initially classified with (N~312) and without (N~100) cognitive impairment according to the FPs are recruited. Equal numbers of patients will be randomly sampled per PCP.

For the evaluation of the implementation process, semi structured interviews and focus groups are held with FPs, practice nurses, key representatives of local dementia services as well as with couples of demented persons and their informal caregivers.

# *A flow chart can be included to give a overview of the procedures that subjects will undergo in the course of research.>*

# STUDY POPULATION

## Population (base)

Cluster RCT: FPs indicated on a list of all their elderly patients (age ≥ 65 years) those patients whom they thought might be cognitively impaired, based on their general impression of patients or their ‘gut feeling’. They could make use of the medical records but did not make use of screening instruments for this classification.

Patients within the primary care practices and their informal carers are eligible for study participation if they fulfil the following criteria:

## Inclusion criteria

1. Patients are classified as possibly cognitively impaired or possibly having dementia based on FPs’ general impression.
2. A primary informal caregiver can be identified. Definition primary informal caregiver: the person who has a central role in (and takes responsibility for) the care of an, independently living, disabled relative or partner.

## Exclusion criteria

1. Patients allready diagnosed with dementia.
2. Patient or (if present) caregiver terminally ill.
3. Permanent admission of the patient to a nursing home expected within 6 months.
4. The patient is not/no longer sufficiently capable of understanding spoken language or expressing him- or herself.

Diagnostic Test Accuracy Study: In order to also get information about the negative predictive value of the FPs cognitive classification, an additonal sample will be drawn of patients FPs classified as ‘no signs of cognitive impairment’ (Figure 2).

The study population for the process evaluation of succes and failure factors of the intervention consists of FPs and practice nurses in the intervention group. In addition, a random selection of patients and caregivers in the intervention group is invited to participate in a semi-structured interview.

## Sample size calculation

In order to come to a sample size that would allow showing significant differences in quality of care, in wellbeing and in numbers of new dementia diagnoses we performed several power calculations:

Quality of care:

The primary outcome is the difference in total score on a set of quality indicators (QIs) after 12 months between intervention and control group. Perry et al. found a mean (crosssectional) concordance to the total set of 45.3% (SD 30.4). A serial measurement is not yet done with this set of QIs.

For this study we expect an increase in concordance in the intervention group of around 1.5 times the baseline scores. In other words; an added 22,5% concordance with the total set of QIs is considered as clinically relevant. We based out powercalculation on the method described by Kerry et al.(Kerry and Bland 1998). To be safe we assumed a somewhat larger within cluster SD of 35 than Perry et al. found. Based on research of Campbell et al on ICCs in primary care research we assumed a between cluster SD of 3(Campbell et al. 2005).

With an α of 0.05 and β of 0.90, a sample size of 5 clusters per arm and 72 patients per arm is required to show a statistically significant and clinically relevant difference in quality of care.

Caregiver wellbeing:

We chose the GHQ12 as a meausure of caregiver mental health as the most important patient and caregiver wellbeing measure. We expect to find results after a period of 12 months on this outcome. We based our sample size for the GHQ 12 on a study of Diaz et al . in 2008. They report a significant difference in GHQ 12 decline between intervention and control group. The average difference in decline was 2.2 points in a period of 6 months. Based on their SDs we (conservatively) estimated SDs in the control- (4.3) and interventiongroup (3.6). Based on the formula: √(SDbaseline^2^ x SDfollow-up^2^).

A paired t-test for average difference with unequal variance shows that assuming an equal distribution over intervention and controlgroup,a desired power of 90%, and an alpha of 0.05 we need to include 57 caregivers per group in order to find significant and clinically relelvant results (SASsoftware). Estimating that about half of patients will have an informal caregiver and that about 20% will be lost to follow-up in the first year and adding 10 % for clustering resulted in a desired sample of 156 patients per arm.

Number of new dementia diagnoses in 12 months:

We based this calculation on a previous study on an educational intervention to improve the number of dementia diagnoses in primary care(Perry et al. ). Perry et al. conducted a cluster RCT design in which they asked 105 FPs to select 5 patients in whom they suspected cognitive impairment. FPs collaborated with a practice nurse in diagnosing dementia. In the intervention group, dementia was diagnosed in 49.1% (130 new diagnoses in 265 patients at risk) of these patients, in the control group in 14.8% (20 new diagnoses in 135 patients at risk) of these patients, after one year. Using the probabilities of Perry et al. and assuming a power of 90%, 20% loss to follow-up and adding 10% for clustering within practices resulted in a desired sample of 55 patients per arm.

Diagnostic accuracy of FPs’ classification at baseline

The diagnostyic accuracy is expressed in the positive and negative predictive value of the FPs at baseline. When we include 312 patients, initially classified by the FPs as cognitive impaired as well as and100 patients initially classified with no cognitive impairment, we reach a confidence interval of 17.9% around the point estimate, assuming a prior positive predictive value of 30%.

# TREATMENT OF SUBJECTS

*intervention studies>*

## Investigational product/treatment

The intervention in this study is based on current evidence. It was developed further in close collaboration with two FPs of our project team and two FPs participating in the study.

The intervention will contain the following elements:

1. Cognitive classification of all elderly patients.

Aimed to increase FPs’ awareness of cognitive dysfunction and dementia in their patients FPs are asked to explicitly classify cognitive function in all their patients above the age of 65, based on their recollection and if needed the medical records. They did a (cross-sectional) classification of the cognitive function of their entire elderly practice population. They could classify patients as: ‘probable dementia’; ‘possible cognitive impairment or dementia’; ‘no signs of cognitive impairment’ or ‘not in sight’. The intervention targets the group classified as possibly cognitively impaired.

During the course of the trial FPs will adjust the classification of patients’ cognitive status when needed. They will record this in the medical records.

2. Training of FPs and practice nurses.

In order to improve diagnosis and management of dementia in primary care, FPs and practice nurses will undergo a training that was part of an effective intervention recently develop by Perry et al.(Perry et al. 2008). The training focuses on collaboration between FPs and practice nurses in diagnosing and managing dementia. FPs will consider barriers to dementia diagnosis and will learn how to diagnose dementia according to current guidelines (particularly the dementia guideline of the Dutch College of General Practitioners). Additionally differential diagnosis and pharmacological treatment of dementia are considered.

In order to improve care for elderly, possibly cognitively impaired patients, practice nurses will learn how to work with the RAI instrument, a standardised instrument for broad functional assessment of elderly patients and their informal caregiver. In addition, they will learn how to make a care plan and evaluate it periodically. The RAI instrument also provides protocols for clinical analyses of identified problems, supporting the formulation of an adequate care plan.

3. The deployment of a practice nurse.

An extra practice nurse who exclusively focuses on the group of patients with possible cognitive impairment is deployed in the intervention practices. She will work with the instruments mentioned above to assess care needs of both the patient and the primary informal caregiver. She will prioritise problems and prepare a care plan in consultation with the informal caregiver. Next, she will share the results with the FPs and finalise the care plan in close collaboration with the FP. For a graphic overview of the intervention see appendix 1.

Additionally, as we expect to identify dementia in a substantial proportion of patients, she will gain expert knowledge on all dementia services in the region and establish close collaboration with secondary care providers. The practice nurse will serve patients of several PCPs. She will be supervised by one of the FPs participating in the study. The practice nurse will have patient contacts according to a predefined schedule; Agreements are made with FPs that they remain responsible for all medical care, including crisis management during the study. Appendix 1 provides an overview of the intervention.

4. Structured collaborative care in the region.

In the region several secondary care providers are involved in dementia care: a) a nursing home recently begun deployment of case managers for home-dwelling dementia patients. b) There is an academic memory clinic and there are two geriatric outpatient clinics that FPs frequently refer to.

We aim to make agreements on collaboration between FPs and these providers based on the outlines for this in the National Collaboration Agreement for primary dementia care. These include for example agreements on information exchange, prescription of drugs, consultation and referral, crisis situations, (crisis-) admission.

The nursing home is planning to organise multidisciplinary expert team meetings. We aim to make agreements about consultation of this expert team by the practice nurses.

**Usual care**

In the usual care group FPs were also asked to explicitly classify cognitive function in all their patients above the age of 65, based on their recollection and if needed the medical records. They will also adjust this classification in the course of the study. No other interventions will be done.

Usual care for the group of patients consists of normal care as provided by FPs.

The literature shows that there is a number of barriers to dementia diagnoses in primary care(Bradford et al. 2009). We therefore expect FPs in the usual care group to be more reluctant to diagnose dementia, additionally because they have less subsequent to offer compared to the intervention group.

When they do proceed to diagnose dementia they will likely do this according to the guideline on dementia of the Dutch College of General Practitioners and when dementia is suspected in the majority of cases FPs will refer patients for further diagnostic evaluation. Follow-up care consists of referral to home care services and possibly more specific services for dementia patients in the region. In general a structured assessment of care needs is not performed nor is a comprehensive care plan made. In a minority of practices, there is a practice nurse for the elderly present. In those practices, structured assessment of care needs of elderly patients and development of a care plan is more likely to occur.

In usual care, there are no explicit agreements on collaboration and referral among care providers in this region.

## Use of co-intervention (if applicable) not applicable

## Escape medication (if applicable) not applicable

##

# INVESTIGATIONAL MEDICINAL PRODUCT

Not applicable

# METHODS

## Study parameters/endpoints

The primary outcome of this study is quality of care. Previous research on the effects of an educational intervention combined with coaching of PCPs and practice nurses demonstrated a positive effect on some elements of quality of care(Perry et al. 2008).

### Main study parameter/endpoint

The primary outcome is quality of care: quality of the diagnostic process and subsequent care is measured by a set of 23 QIs (Perry et al. 2010). **Appendix 2** provides an overview of these QIs.

### Secondary study parameters/endpoints (if applicable)

Patient and caregiver wellbeing and cost-effectiveness:

**Appendix 3** provides an overview of caregiver and patient outcomes and potential effect modifiers measured. There is no information on cost-effectiveness of this type of interventions. Therefore we will also measure health care utilisation of both the patient and the caregiver, including time till institutionalisation of the person with dementia. In order to assess a long term effect we will follow patients and their informal caregivers for the period of three years.

Diagnostic accuracy study: the FPs cognitive classifications ‘possible cognitive impairment/dementia’ and ‘no signs of cognitive impairment’ are compared to the CAMCOG diagnosis on the presence or absence of dementia or MCI.

Feasibility of the intervention:

The level of implementation will be measured. For this the PN will record all her activities in a diary; additionally medical records are used.

Success and failure factors of the intervention at different levels will be derived from (2) focus groups with FPs and PNs. Additionally semi-structured interviews will be conducted with 5 persons with dementia and their informal caregiver, in order to also include the patient perspective.

### Other study parameters (if applicable)

## Randomisation, blinding and treatment allocation

We will use prognostic stratification before randomisation. Each primary care practice will be stratified based on the two most important potential effect modifiers. Based on previous research of Perry et al. these are a) whether FPs collaborate with a practice nurse and b) the percentage of elderly patients within practices.

**Figure 2** provides an overview of the patient flow of the cluster RCT.

## Study procedures

Subjects will not undergo any new or experimental treatment in the course of this study. Their providers will be trained to deliver care (both the process of diagnosis, disclosure and psychoeducation and the process of follow-up care management and support) in closer concordance with current guidelines. Care will become more structured both at the level of the PCP and within the region. For the subjects this will presumably result in an earlier and more thorough assessment of their care needs and earlier intervention or referral. No subjects will be withheld from treatment. We aim to introduce the intervention also in the control group. Because of feasibility the intervention will be introduced in this group one year later.

*Eligibility*

The researchers will assess eligibility of all patients for the study that were diagnosed with dementia. Before approaching potential participants, FPs will go through the list of potential participants to see whether there are any other contraindications, besides the predefined exclusion criteria, to study participation of these persons.

*Recruitment*

Subjects will be informed about the study by means of an invitational letter from their FP and an information leaflet with detailed information about the study: aim of the study, what participation means for participants, freedom to not participate or to end participation at any time without having to give a reason, etc. Subjects are encouraged to discuss the matter with friends or relatives or with their FP. Some subjects may not want to be contacted about the study. They can communicate this by returning a form by post: on this form subjects can indicate that they are not interested to participate in the study and that they do not want to be contacted. A return envelope is provided. (letter and information leaflet are added to this protocol as separate documents)

If subjects do not object, they are contacted by telephone by one of the researchers. Additional clarification about the study is provided, if needed. Subjects are asked whether they are interested to participate in the study. If this is so, an appointment will be made for obtaining informed consent and for the baseline interview.

*Informed consent*

Informed consent will be obtained for participation in the questionnaire and interview part of the study. Informed consent is obtained both from the person with possible cognitive impairment as from the caregiver. If persons are not capable to give informed consent themselves, a legal attorney will be asked to give informed consent on their behalf. People with possible cognitive impairment are considered incapable of giving informed consent if they are not capable to reproduce the goal of the study and what it would mean for them to participate.

*Interviews and questionnaire*

Structured interviews are conducted by trained interviewers. For this patients and caregivers will be visited at home half yearly. The questionnaires for informal caregivers are posted one week in advance to the home visit for the interview. Caregivers are asked to fill in the questionnaire within this period so that the interviewer can collect the questionnaire when visiting.

*Care utilisation diary*

Caregivers are asked to record the formal and informal care use of the one they are caring for. This will be checked and if necessary supplemented during the 6 monthly interviews.

*Process evaluation*

*General approach:* We apply the approach for process evaluations for implementation of care innovations of Meiland et al. 2005. Meiland’s approach of adaptive implementation distinguishes (external) factors that throughout the process affect the implementation such as characteristics of the intervention, preconditions for the intervention, human and financial resources. Also during the various stages of implementation various factors can play a role, namely during preparation, the introduction and the continuation phase. Within each phase distinction is made between micro level (patient / caregiver and individual worker), meso level (between and within care organizations) and macro level (legislation, financing context).

To give an impression at the micro level questions will be asked such as:
(i) To what extent are the health needs identified early and properly diagnosed according to carers? (ii) To what extent can adequately respond to identified health needs? (iii) Has the coordinated dementia care a value according to the user?
The exact number of interviews is determined by the point of saturation of information is reached (saturation). The interviews are recorded, analyzed and uncertainties are checked with the respondents for validation.

*Practical approach:* To gain more insight in the feasibility of the intervention at the level of the primary care practice and in the context of regional elderly/dementia care providers, discussions with FPs and local care providers will be organized. Additionally a focus group with FPs and practice nurses will be held during the course of the project in order to further explore success and failure factors of the intervention. To get insight into failure and success factors on a regional level in addition key representatives of local care providers/services are interviewed.

We will also conduct semi-structured interviews with 5 random couples of persons with possible cognitive impairment and their informal caregivers in order to get a view of their experiences with the new form of care.

*Analytical approach:* The interviews are transcribed verbatim and all material is written using a qualitative analysis program analysis. The texts are encoded by two independent researchers based on the theoretical model. Differences in coding between the researchers discussed until agreement is reached. Then the per code excerpts and summarized in matrices described (Miles & Huberman, 1994). On this basis, facilitating and impeding factors for the introduction of integrated care and empowerment are described and recommendations for further implementation activities.

**Figure 1: timeline development, cluster RCT and qualitative evaluation**

**2010/11**

Preparation & Randomisation PCPs (clusters)

**2011** control group

Intervention group

**2012** intervention group

Development intervention including focus group FPs (intervention group)

Focus group with FPs and PNs to evaluate success and failure factors of the intervention

Semi-structured interviews with 5 persons with cognitive impairment and their caregiver

**Figure 2: patient flow cluster RCT (goals 1-3) & diagnostic accuracy sub-study (goal 4)**

**All patients ≥ 65**

N = 7798 patients, 16 PCPs

**Patients with possible cognitive impairment according to FPs**

N = 650

**Intervention group**

8 PCPs

**1. Random sample**

N = 312

**Control group**

8 PCPs

- Outcomes in patients with a baseline diagnosis MCI or dementia on the CAMCOG and their informal caregiver

(n ~ 156)

- Patient and caregiver characteristics and outcomes (n =156)

- CAMCOG (n =156)

- CAMCOG (n = 156)

- Quality of care indicators, diagnostic (N=156) & management indicators in patients with dementia (n ~ 50)

**Measurement**

- Patient and caregiver characteristics and outcomes (n =156)

- CAMCOG (n =156)

- Outcomes in patients with a baseline diagnosis MCI or dementia on the CAMCOG and their informal caregiver

(n ~ 156)

**T0 Baseline**

**6 months**

**12 months**

**18 months**

**24 months**

**12 months**

- CAMCOG (n = 156)

- Quality of care indicators, diagnostic (N=156) & management indicators in patients with dementia (n ~ 50)

**2. Random sample, stratified on age, gender, GP (to mirror sample 1)** N=100

**Patients without cognitive impairment according to FPs**

N = 7148

- CAMCOG

Demografie

ADL, Chr zkt, contact freq huisarts

(n =100)

## Withdrawal of individual subjects

Study participants can end their participation at any point in time without having to give any reason for this. This will not in any way affect the care they receive.

## Replacement of individual subjects after withdrawal

Participants withdrawing from the study will not be replaced; we will include a closed cohort.

## Follow-up of subjects withdrawn from treatment

We will try to find out what the reason of withdrawal was by offering subjects who have withdrawn a short telephone interview. If they do not whish to collaborate in this, this will of course be respected.

## Premature termination of the study

< *Please describe the criteria for terminating the study prematurely and the procedures in case the study will be terminated prematurely.*>

# SAFETY REPORTING

## Section 10 WMO event

In accordance to section 10, subsection 1, of the WMO, the investigator will inform the subjects and the reviewing accredited METC if anything occurs, on the basis of which it appears that the disadvantages of participation may be significantly greater than was foreseen in the research proposal. The study will be suspended pending further review by the accredited METC, except insofar as suspension would jeopardise the subjects’ health. The investigator will take care that all subjects are kept informed.

## Adverse and serious adverse events

< *Please describe the procedures for handling the adverse and serious adverse events. If certain SAEs do not require immediate reporting, please specify*.>

Adverse events are defined as any undesirable experience occurring to a subject during a clinical trial, whether or not considered related to the investigational drug. All adverse events reported spontaneously by the subject or observed by the investiga­tor or his staff will be recorded.

A serious adverse event is any untoward medical occurrence or effect that at any dose results in death;

- is life threatening (at the time of the event);
- requires hospitalisation or prolongation of existing inpatients’ hospitalisation;
- results in persistent or significant disability or incapacity;
- is a congenital anomaly or birth defect;
- is a new event of the trial likely to affect the safety of the subjects, such as an unexpected outcome of an adverse reaction, lack of efficacy of an IMP used for the treatment of a life threatening disease, major safety finding from a newly completed animal study, etc.

All SAEs will be reported to the accredited METC that approved the protocol, according to the requirements of that METC.

### Suspected unexpected serious adverse reactions (SUSAR)

<C*hapters 8.2.1 and 8.2.2 are only applicable for studies with an investigational medicinal product.*

<*Please describe also the method of breaking the code for SUSAR reporting*.>

Adverse reactions are all untoward and unintended responses to an investigational product related to any dose administered.

Unexpected adverse reactions are adverse reactions, of which the nature, or severity, is not consistent with the applicable product information (e.g. Investigator’s Brochure for an unapproved IMP or Summary of Product Characteristics (SPC) for an authorised medicinal product).

The sponsor will report expedited the following SUSARs to the METC:

- SUSARs that have arisen in the clinical trial that was assessed by the METC;
- SUSARs that have arisen in other clinical trial of the same sponsor and with the same medicinal product, and that could have consequences for the safety of the subjects involved in the clinical trial that was assessed by the METC.

The remaining SUSARs are recorded in an overview list (line-listing) that will be submitted once every half year to the METC. This line-listing provides an overview of all SUSARs from the study medicine, accompanied by a brief report highlighting the main points of concern.

The sponsor will report expedited all SUSARs to the competent authority, the Medicine Evaluation Board and the competent authorities in other Member States.

< *SUSARs that are already reported to the EMEA Eudravigilance database do not have to be once again reported to the competent authority and the MEB because they have direct access to the Eudravigilance database.*>

The expedited reporting will occur not later than 15 days after the sponsor has first knowledge of the adverse reactions. For fatal or life threatening cases the term will be maximal 7 days for a preliminary report with another 8 days for completion of the report.

### Annual safety report

< *The annual safety report may be combined with the annual progress report (see chapter 12.3)*.>

In addition to the expedited reporting of SUSARs, the sponsor will submit, once a year throughout the clinical trial, a safety report to the accredited METC, competent authority, Medicine Evaluation Board and competent authorities of the concerned Member States.

This safety report consists of:

- a list of all suspected (unexpected or expected) serious adverse reactions, along with an aggregated summary table of all reported serious adverse reactions, ordered by organ system, per study;
- a report concerning the safety of the subjects, consisting of a complete safety analysis and an evaluation of the balance between the efficacy and the harmfulness of the medicine under investigation.

## Follow-up of adverse events

All adverse events will be followed until they have abated, or until a stable situation has been reached. Depending on the event, follow up may require additional tests or medical procedures as indicated, and/or referral to the general physician or a medical specialist.

## Data Safety Monitoring Board (DSMB)

<*In case a DSMB is established to perform ongoing safety surveillance and to perform interim analyses on the safety data, this committee should be an independent committee. The composition of DSMB should be described and it should be clear that each member has no conflict of interest with the sponsor or company of the study*.>

<*Criteria on which the DSMB may decide to terminate the trial prematurely should be clearly defined before the trial has started.*>

# STATISTICAL ANALYSIS

## Descriptive statistics

The diagnostic accuracy of the FPs will be described (goal 4).

## Univariate analysis

Not applicable

## Multivariate analysis

We will do multilevel multivariate analysis to compare the two care conditions controlling for baseline imbalance and clustering effects within practices on number of incident diagnoses (goal 1), quality of care (goal 2) patient and caregiver outcomes (goal 3). Effect modification will be explored on principal characteristics (age, gender, severity dementia and type of caregiver). A post hoc analysis on main outcomes will be done on patients who score under critical cut off on CAMCOG at baseline (<86).

In addition, an economic evaluation is performed from societal perspective comparing the effects of goals 1 to 3 and costs in the two conditions. To compare costs between the two groups, confidence intervals for the differences in mean costs are calculated using bias-corrected and accelerated bootstrapping with 5000 replications. For the cost-effectiveness analysis the difference in total costs between the intervention and usual care group are compared with the difference in main clinical outcomes.

## Interim analysis (if applicable)

Not applicable

# ETHICAL CONSIDERATIONS

## Regulation statement

<*In this section it can be stated that the study will be conducted according to the principles of the Declaration of Helsinki (version, date, see for the most recent version: www.wma.net) and in accordance with the Medical Research Involving Human Subjects Act (WMO) and other guidelines, regulations and Acts (if applicable, please specify)*>

## Recruitment and consent

Please see chapter 7.3 Study procedures.

## Objection by minors or incapacitated subjects (if applicable)

Not applicable

## Benefits and risks assessment, group relatedness

<*Please give a justification of the proposed study.*>

## Compensation for injury

The sponsor/investigator has a liability insurance which is in accordance with article 7, subsection 6 of the WMO.

The sponsor (also) has an insurance which is in accordance with the legal requirements in the Netherlands (Article 7 WMO and the Measure regarding Compulsory Insurance for Clinical Research in Humans of 23th June 2003). This insurance provides cover for damage to research subjects through injury or death caused by the study.

1. € 450.000,-- (i.e. four hundred and fifty thousand Euro) for death or injury for each subject who participates in the Research;
2. € 3.500.000,-- (i.e. three million five hundred thousand Euro) for death or injury for all subjects who participate in the Research;
3. € 5.000.000,-- (i.e. five million Euro) for the total damage incurred by the organisation for all damage disclosed by scientific research for the Sponsor as ‘verrichter’ in the meaning of said Act in each year of insurance coverage.

The insurance applies to the damage that becomes apparent during the study or within 4 years after the end of the study.

## Incentives (if applicable)

FPs get accreditation points for their participation in the training. The practice nurse is initially paid out of study resources. The goal is to increasingly draw on normal primary care resources (health insurance etc.) in the course of the project.

# ADMINISTRATIVE ASPECTS AND PUBLICATION

## Handling and storage of data and documents

All data is gathered either at the PCPs or at subjects’ homes (interviews/questionnaires). Data is transported on USB-sticks only. Coded data files may also be send by email. Data is stored on a hard disk of the research institute that is only accessible for authorised persons (only members of the research team are authorised) with a username and password. All data will be coded. Coding will be based on village, PCP, FP and a random number for subjects. There is no relation with initials or birth-date. Data will be stored for no longer than 115 years. The key to the code will be safeguarded by the principal investigator (HvH).

## Amendments

Amendments are changes made to the research after a favourable opinion by the accredited METC has been given. All amendments will be notified to the METC that gave a favourable opinion.

## Annual progress report

The sponsor/investigator will submit a summary of the progress of the trial to the accredited METC once a year. Information will be provided on the date of inclusion of the first subject, numbers of subjects included and numbers of subjects that have completed the trial, serious adverse events/ serious adverse reactions, other problems, and amendments.

## End of study report

The investigator will notify the accredited METC of the end of the study within a period of 8 weeks. The end of the study is defined as the last patient’s last visit.

In case the study is ended prematurely, the investigator will notify the accredited METC, including the reasons for the premature termination.

 Within one year after the end of the study, the investigator/sponsor will submit a final study report with the results of the study, including any publications/abstracts of the study, to the accredited METC.

## Public disclosure and publication policy

<*Please mention the arrangements made between the sponsor and the investigator concerning the public disclosure and publication of the research data. >*

# Appendices

**Appendix 1. Overview intervention**

**Possible cognitive impairment**

Screening PN

Consultation FP

**Dementia or MCI suspected?**

RAI Contact Assessment

MMSE, VAT, GDS, gehoor, visus.

b) Physical & neurological examination

c) Medication review

d) Diagnosis and disclosure

e) Diagnostic referral?

- Psychoeducation and support

- RAI Home Care if indicated by RAI Contact Assessment or if dementia

- Care plan

- Referral to services

**Yes**

**Revision care plan every 6 months or earlier when required**

**Dementia or MCI diagnosis?**

**Yes**

**No**

NHG-guideline recommendations:

a) Laboratory tests

Assessment and care PN

**Revision screening PN after 6 months**

**PN = practice nurse, RAI = Resident Assessment Instrument (comprehensive care needs assessment instrument) VAT = Visual Analogue Test, MMSE = Mini Mental State Examination, GDS = Geriatric Depression Scale**

**Appendix 2. overview of QIs, measured at baseline and at 12 months**

| **Process indicators (diagnosis)** |
| --- |
| 1. IF signs of dementia are noticed, THEN the general practitioner should start cognitive diagnostic workup within 3 months |
| 2. IF cognitive diagnostic workup has been started, THEN a ‘‘history from other sources’’ should be obtained. |
| 3. IF cognitive diagnostic workup has been started, THEN general physical examination should be conducted. |
| 4. IF cognitive diagnostic workup has been started, THEN neurological examination should be conducted. |
| 5. IF cognitive diagnostic workup has been started, THEN a blood test (at least erythrocyte  sedimentation rate, hemoglobin, mean corpuscular volume, creatinine, thyroidstimulating hormone, glucose) should be conducted. |
| 6. IF cognitive diagnostic workup has been started, THEN a cognitive screening test should be conducted. |
| 7. IF cognitive diagnostic workup has been started, THEN a mood screening test should be conducted. |
| 8. IF cognitive diagnostic workup has been started, THEN the patient’s physical, emotional and social needs should be assessed. |
| 9. IF cognitive diagnostic workup has been started, THEN the caregiver’s burden and needs should be assessed. |
| 10. IF a dementia diagnosis has been made, THEN it should meet the DSM-IV criteria.  1. Memory disorder  2. Other cognitive function disorder  3. Negative influence on daily functioning  4. Absence of a delirium |
| 11. IF a diagnosis has been made after cognitive diagnostic workup, THEN the general practitioner should disclose the diagnosis to patient and caregiver. |
| 12. IF a patient meets one of the following criteria, THEN the general practitioner should refer the patient to a multidisciplinary memory clinic.  1. Uncertainty about the diagnosis  2. Dementia diagnosis was made, and patients showed the following characteristics.  a. The course of the dementia was stepwise or rapidly progressive.  b. Characteristics that suggest rare types of dementia, like focal of frontal features or visual hallucinations in early stages of the dementia  c. Symptoms or results that suggest disorders that can only be treated by a specialist  d. Patient younger than 65  3. Patients or caregivers wish to start pharmacological treatment to ease dementia symptoms (cholinesterase inhibitors)  4. Patient’s or caregiver’s request to confirm the diagnosis |
| 13. IF a dementia diagnosis has been made, THEN the general practitioner should formally bring the cognitive evaluation to a close, reviewing the following items.  1. Explicit disclosure of the diagnosis  2. Information about the prognosis  3. Offering leaflets, written information on dementia  4. Suggestions on home care  5. Information on possibilities of cholinesterase inhibitor treatment in dementia  6. Driving abilities |
| **Process indicators (follow-up)** |
| 14. IF a dementia diagnosis has been made, THEN an individualized care plan for the patient should be formulated. |
| 15. IF a dementia diagnosis has been made, THEN an individualized care plan for the caregiver should be formulated. |
| 16. IF a dementia diagnosis has been made, THEN the care plan should be evaluated and if necessary updated every 3 months. |
| **Structure indicators** |
| 17. Cognitive diagnostic examination consists of at least two face-to-face consultations. |
| 18. The general practitioner is the first contact point or will delegate this task to the practice nurse. |
| 19. A guidebook, listing all social services for dementia patients and their caregivers, is available in the family practice. |
| 20. General practitioners and practice nurses use the same medical record. |
| **Outcome indicators** |
| 21. Continuity of care (indicators 16, 19, AND 21 complied with = 16, 18, 20?) |
| 22. Number of emergency consultations in dementia patients |
| 23. Satisfaction of the patient and the informal caregiver was assessed, using a validated questionnaire |

**Appendix 3. Overview of caregiver and patient outcomes and potential effect modifiers**

| *OVERVIEW* | Instrument | Items | Form | Time min | T0 | T1 | T2 | T3 | T4 |
| --- | --- | --- | --- | --- | --- | --- | --- | --- | --- |
| *Outcomes caregiver* |  |  |  |  |  | 6 m | 12m | 18m | 24m |
| Burden | SSCQ | 12 | I | 10 | X | X | X | X | X |
| QoL | MDS* & EQ5D |  | Q | 20 | X | X | X | X | X |
| Psychopathology | GHQ12 | 12 | I | 10 | X |  | X |  | X |
| Care utilisation, care time and absenteeism | MDSe* / cost diary |  | Q/I | 15 | X | X | X | X | X |
| *Outcomes pt* |  |  |  |  |  |  |  |  |  |
| Presence of dementia / MCI | CAMCOG^1^ |  | I | 25 | X |  | X |  |  |
| QoL | QoL-AD & EQ5D | 16 | I | 15 | X | X | X | X | X |
| Care utilisation etc. | MDSe* / cost diary by porxy |  | Q/I | 0 | X | X | X | X | X |
| Time until nursing home admission | According to carer or PCP | - | I |  |  |  | X |  | X |
| *Potential effect-modifiers / confounders* |  |  |  |  |  |  |  |  |  |
| Carer: |  |  |  |  |  |  |  |  |  |
| Sociodemography | MDS | 5 | I |  | X |  |  |  |  |
| Social support  Duration and intensity of caring | SSSL  Added Q | 8  1  1 | Q  I |  | X  X |  | X |  | X |
| Patient: |  |  |  |  |  |  |  |  |  |
| Sociodemography | By proxy | 5 | I | 5 | X |  |  |  |  |
| Dementia type | Medical Records | 1 | I | 0 |  |  | X |  |  |
| Morbidity | MDS by proxy | 1 | I | 5 | X |  |  |  |  |
| Behavioural symptoms | NPI | 12 | I | 10 | X |  | X |  | X |
| PCP / primary care practice:  - Age  - Sex  - Presence of practice nurse for elderly patients  - Interested in dementia  - % patients ≥ 65  - Cluster size | Q  Q  Added Q  Added Q  Medical rec | 1  1  1  3 | Q  Q  Q  Q |  | X  X  X  X  X  X |  |  |  |  |

I=Interview

Q=Questionnaire

MDS* / MDSe*: Minimal DataSet / Minimal DataSet cost-effectiveness respectively (NPO-ZonMW).

^1^ intervention arm only

# REFERENCES

<*Include all key references published in peer reviews journals that are relevant for the study and are discussed in the protocol. Do make sure that the references are up to date.*>

Reference List

1. Ferri CP, Sousa R, Albanese E, Ribeiro WS, Honyashiki M. 2009. World Alzheimer Report 2009. In .

2. Boise L, Neal MB, Kaye J. 2004. Dementia assessment in primary care: results from a study in three managed care systems. *J Gerontol A Biol Sci Med Sci* **59**(6): M621-M626.

3. Sandholzer H, Breull A, Fischer GC. 1999. [Early diagnosis and early treatment of cognitive disorders: a study of geriatric screening of an unselected patient population in general practice]. *Zeitschrift fur Gerontologie und Geriatrie* **32**(3): 172-178.

4. Boise L, Neal MB, Kaye J. 2004. Dementia assessment in primary care: results from a study in three managed care systems. *J Gerontol A Biol Sci Med Sci* **59**(6): M621-M626.

5. Meerman L, van de Lisdonk EH, Koopmans RTCM, Zielhuis GA, Olde Rikkert MGM. 2008. Prognosis and vascular co-morbidity in dementia a historical cohort study in general practice. *J Nutr Health Aging* **12**(2): 145-150.

6. Bradford A, Kunik M, Schulz P, Williams S, Singh H. 2009. Missed and Delayed Diagnosis of Dementia in Primary Care: Prevalence and Contributing Factors. *Alzheimer Dis Assoc Disord* .

7. Downs M. 2006. Dementia: Diagnosis and management in primary care - a primary care based education/research project. *ISRCTN Register* .

8. Perry M, Draskovic I, van Achterberg T, Borm GF, van Eijken MIJ, Lucassen P, Vernooij-Dassen MJFJ, Olde Rikkert MGM. 2008. Can an EASYcare based dementia training programme improve diagnostic assessment and management of dementia by general practitioners and primary care nurses? The design of a randomised controlled trial. *BMC Health Serv Res* **8**: 71.

9. Perry et al. Submitted. In .

10. Hinton L, Franz CE, Reddy G, Flores Y, Kravitz RL, Barker JC. 2007. Practice constraints, behavioral problems, and dementia care: primary care physicians' perspectives. *J Gen Intern Med* **22**(11): 1487-1492.

11. Wenger NS, Solomon DH, Roth CP, MacLean CH, Saliba D, Kamberg CJ, Rubenstein LZ, Young RT, Sloss EM, Louie R, Adams J, Chang JT, Venus PJ, Schnelle JF, Shekelle PG. 2003. The quality of medical care provided to vulnerable community-dwelling older patients. *Annals of Internal Medicine* **139**(9): 740-747.

12. Van Hout H, Vernooij-Dassen M, Bakker K, Blom M, Grol R. 2000. General practitioners on dementia: tasks, practices and obstacles. *Patient Educ Couns* **39**(2-3): 219-225.

13. Schoenmakers B, Buntinx F, Delepeleire J. 2009. What is the role of the general practitioner towards the family caregiver of a community-dwelling demented relative? A systematic literature review. *Scand J Prim Health Care* **27**(1): 31-40.

14. Hinton L, Franz CE, Reddy G, Flores Y, Kravitz RL, Barker JC. 2007. Practice constraints, behavioral problems, and dementia care: primary care physicians' perspectives. *J Gen Intern Med* **22**(11): 1487-1492.

15. Yaffe MJ, Orzeck P, Barylak L. 2008. Family physicians' perspectives on care of dementia patients and family caregivers. *Can Fam Physician* **54**(7): 1008-1015.

16. Cody M, Beck C, Shue VM, Pope S. 2002. Reported practices of primary care physicians in the diagnosis and management of dementia. *Aging & mental health* **6**(1): 72-76.

17. Vickrey BG, Mittman BS, Connor KI, Pearson ML, la Penna RD, Ganiats TG, Demonte RWJ, Chodosh J, Cui X, Vassar S, Duan N, Lee M. 2006. The effect of a disease management intervention on quality and outcomes of dementia care: a randomized, controlled trial. *Annals of Internal Medicine* **145**(10): 713-726.

18. Reuben DB, Roth C, Kamberg C, Wenger NS. 2003. Restructuring primary care practices to manage geriatric syndromes: the ACOVE-2 intervention. *J Am Geriatr Soc* **51**(12): 1787-1793.

19. Reuben DB, Roth CP, Frank JC, Hirsch SH, Katz D, McCreath H, Younger J, Murawski M, Edgerly E, Maher J, Maslow K, Wenger NS. 2010. Assessing care of vulnerable elders--Alzheimer's disease: a pilot study of a practice redesign intervention to improve the quality of dementia care. *J Am Geriatr Soc* **58**(2): 324-329.

20. Cody M, Beck C, Shue VM, Pope S. 2002. Reported practices of primary care physicians in the diagnosis and management of dementia. *Aging & mental health* **6**(1): 72-76.

21. Van Hout H, Vernooij-Dassen M, Bakker K, Blom M, Grol R. 2000. General practitioners on dementia: tasks, practices and obstacles. *Patient Educ Couns* **39**(2-3): 219-225.

22. Bryans M, Keady J, Turner S, Wilcock J, Downs M, Iliffe S. 2003. An exploratory survey into primary care nurses and dementia care. *British journal of nursing (Mark Allen Publishing)* **12**(17): 1029-1037.

23. Callahan CM, Boustani MA, Unverzagt FW, Austrom MG, Damush TM, Perkins AJ, Fultz BA, Hui SL, Counsell SR, Hendrie HC. 2006. Effectiveness of collaborative care for older adults with Alzheimer disease in primary care: a randomized controlled trial. *JAMA* **295**(18): 2148-2157.

24. Chenoweth L, King MT, Jeon YH, Brodaty H, Stein-Parbury J, Norman R, Haas M, Luscombe G. 2009. Caring for Aged Dementia Care Resident Study (CADRES) of person-centred care, dementia-care mapping, and usual care in dementia: a cluster-randomised trial. *Lancet Neurol* **8**(4): 317-325.

25. Kerry SM, Bland JM. 1998. Sample size in cluster randomisation. *BMJ* **316**(7130): 549.

26. Campbell MK, Fayers PM, Grimshaw JM. 2005. Determinants of the intracluster correlation coefficient in cluster randomized trials: the case of implementation research. *Clin Trials* **2**(2): 99-107.

27. Perry et al. Submitted. In .

28. Bradford A, Kunik M, Schulz P, Williams S, Singh H. 2009. Missed and Delayed Diagnosis of Dementia in Primary Care: Prevalence and Contributing Factors. *Alzheimer Dis Assoc Disord* .

29. Perry M, Draskovic I, van Achterberg T, van Eijken M, Lucassen P, Vernooij-Dassen M, Olde Rikkert M. 2010. Development and validation of quality indicators for dementia diagnosis and management in a primary care setting. *Journal of the American Geriatrics Society* **58**(3): 557-563.
